# The role of adjuvant chemotherapy in rectal cancer patients with ypT0-2N0 after neoadjuvant chemoradiotherapy

**DOI:** 10.3389/fonc.2024.1338098

**Published:** 2024-02-09

**Authors:** Jianguo Yang, Qican Deng, Zhenzhou Chen, Yajun Chen, Zhongxue Fu

**Affiliations:** Department of General Surgery, The Third Affiliated Hospital of Chongqing Medical University, Chongqing, China

**Keywords:** rectal cancer, neoadjuvant chemoradiotherapy, tumor response, adjuvant chemotherapy, oncological outcome

## Abstract

**Background:**

Neoadjuvant chemoradiotherapy has emerged as the established treatment for locally advanced rectal cancer. Nevertheless, there remains a debate regarding the necessity of adjuvant chemotherapy for patients with locally advanced rectal cancer who exhibit a favorable tumor response (ypT0-2N0) after neoadjuvant chemoradiotherapy and surgery. Thus, the objective of this study is to investigate the impact of adjuvant chemotherapy on the oncological prognosis of rectal cancer patients who have a good response to neoadjuvant chemoradiotherapy.

**Materials and methods:**

The study was conducted following the Preferred Reporting Items for Systematic Reviews and Meta-Analyses protocol. Articles were searched in the Web of Science, PubMed, and Cochrane Library databases. The primary outcomes assessed were 5-year overall survival, disease-free survival, cancer-specific survival, recurrence-free survival, local recurrence, and distant metastasis. The data was summarized using a random effects model.

**Results:**

A meta-analysis was conducted using 18 retrospective studies published between 2009 and 2023. The studies included 9 from China and 5 from Korea, involving a total of 6566 patients with ypT0-2N0 rectal cancer after neoadjuvant chemoradiotherapy. The pooled data revealed that adjuvant chemotherapy significantly improved 5-year overall survival (OR=1.75, 95% CI: 1.15-2.65, P=0.008), recurrence-free survival (OR=1.73, 95% CI: 1.20-2.48, P=0.003), and reduced distant metastasis (OR=0.68, 95% CI: 0.51-0.92, P=0.011). However, adjuvant chemotherapy did not have a significant effect on disease-free survival, cancer-specific survival, and local recurrence in ypT0-2N0 rectal cancer. Subgroup analysis indicated that adjuvant chemotherapy was beneficial in improving overall survival for ypT1-2N0 rectal cancer (OR=1.89, 95% CI: 1.13-3.19, P=0.003).

**Conclusion:**

The findings of the meta-analysis suggest that adjuvant chemotherapy may provide benefits in terms of oncological outcomes for rectal cancer patients with ypT0-2N0 after neoadjuvant chemoradiotherapy and radical surgery. However, further prospective clinical studies are needed to confirm these findings.

## 1 Introduction

Colorectal cancer (CRC) has emerged as the third most common cancer and the second leading cause of cancer-related deaths, according to the latest cancer statistics. The incidence and mortality rates of CRC continue to rise rapidly. Notably, rectal cancer constitutes a significant proportion of all CRC cases (1, 2). Locally advanced rectal cancer (LARC), comprising 50% to 70% of rectal cancer cases, is typically treated with neoadjuvant chemoradiotherapy (NCRT) followed by total mesorectal excision (TME). NCRT not only reduces the size of the tumor, eliminates potential micro metastases, and lowers the risk of local recurrence, but also improves the rate of negative circumferential resection margin of specimen and sphincter-preservation (3). Despite these advancements in treatment, approximately 30% of patients with LARC still experience distant metastasis, which remains the primary cause of cancer-related deaths.

Adjuvant chemotherapy (ACT) is commonly employed after radical operation to eliminate circulating tumor cells and micro metastases, thereby reducing the risk of distant metastasis (4, 5). According to the National Comprehensive Cancer Network (NCCN) guidelines, patients who undergo NCRT should receive oxaliplatin-based ACT after radical surgery, irrespective of tumor response (6). However, there is still ongoing debate regarding the necessity of ACT after surgery following NCRT. A meta-analysis of published studies revealed that ACT improved overall survival (OS) and disease-free survival (DFS) in patients with LARC (7). The EORTC 22921 trial indicated that ACT with 5-fluorouracil and leucovorin for four cycles after neoadjuvant radiotherapy or chemoradiotherapy significantly decreased the risk of local recurrence in rectal cancer patients (8). However, the long-term benefits of ACT in terms of OS and DFS were not observed during a 10-year follow-up (9). A meta-analysis of four randomized controlled trials (RCTs) suggested that 5-fluorouracil-based ACT did not improve OS, DFS, and distant recurrence in rectal cancer patients after NCRT (10). The European Rectal Cancer Consensus Conference (EURECA-CC2) also highlighted the lack of concrete evidence supporting the effectiveness of ACT for oncological outcomes in rectal cancer after NCRT (11).

The benefit of ACT in patients with rectal cancer may vary depending on the pathological T-stage or lymph node status of the tumor. Several studies have shown that ACT improves DFS and OS by 5%-25% in rectal cancer patients with local lymph node metastases (12–16). Additionally, studies have also indicated that rectal cancer patients who exhibit downstaging to ypT0-2N0 after NCRT have favorable oncological outcomes (17–20). However, there is controversy surrounding the use of ACT in rectal cancer patients who respond well (ypT0-2N0) to NCRT. A phase III randomized controlled trial revealed that postoperative chemotherapy significantly enhanced OS in rectal cancer patients with ypT0-2, but there was no evidence to suggest that ACT was beneficial for survival in ypT3-4 stage rectal cancer (21). It should be noted that not all patients with ypT0-2N0 rectal cancer may benefit from ACT (22, 23). Therefore, we conducted this meta-analysis to explore the impact of ACT on the oncological outcomes of rectal cancer patients who demonstrated a good response (ypT0-2N0) to NCRT.

## 2 Materials and methods

This study followed the preferred reporting items for systematic reviews and meta-analyses (PRISMA) guidelines to ensure the feasibility and integrity of the meta-analysis (24) (**Supplementary Table 1**).

### 2.1 Literature search

A comprehensive literature search was conducted by two investigators in PubMed, Web of Science, and Cochrane Library databases. The search period spanned from the establishment of the database to October 1, 2023. The search keywords were set to find the studies on the effect of adjuvant therapy on the oncological outcome of rectal cancer patients treated with NCRT or radiotherapy. The keywords were follows: ((“ neoadjuvant “or” preoperative “) and (“ chemoradiotherapy “or” treatment “or” radiotherapy “or” treatment “) and ((“ rectal cancer “or” postoperative “). And (“ rectal cancer “or” rectal cancer “or” rectal tumor “) and (“ adjuvant “or” postoperative “) and (“ chemotherapy “or” treatment “)). After retrieving the relevant literature, the reviewers screened the studies based on their titles and abstracts, and thoroughly reviewed the full text. Additionally, the researchers supplemented the search by considering the references of the included studies for potentially eligible literature.

### 2.2 Eligibility criteria

The inclusion criteria encompassed: (1) Subjective: patients with primary rectal cancer who underwent neoadjuvant radiotherapy or chemoradiotherapy and TME surgery (abdominoperineal resection, anterior resection, Hartmann procedure, and intersphincteric resection), with good response (ypT0-2N0). (2) Interventions: ACT or observation were performed following NCRT and TME. (3) Type of outcome: the study focused on various oncological outcomes, including OS, DFS, recurrence-free survival (RFS), cancer-specific survival (CSS), local recurrence, and distant metastasis. (4) Type of study: the eligible studies included RCTs and retrospective cohort studies.

The exclusion criteria involved studies that solely performed local excision or neoadjuvant chemotherapy, studies where data on oncological outcomes could not be extracted, studies reporting only on rectal cancer patients with pathological complete responses (pCR) and ypT0-2Nx, and abstracts, meta-analyses, reviews, comments, and letters.

LARC was defined as cT3/4, N0, M0 or cTx, N1-2, M0 rectal cancer at initial diagnosis. pCR was defined as the absence of tumor cells in the primary tumor and lymph nodes after neoadjuvant therapy (ypT0N0M0). DFS was defined as the time from the date of surgery to the detection of disease relapse or death. RFS was defined as the time from the date of surgery to disease relapse (local or distant metastases). OS was defined as the time from the date of surgery to the date of death from any cause. CSS was defined as the time from the date of surgery to death caused by tumor progression. The assessment of prevention for distant metastasis and local recurrence is typically based on regular follow-up examinations, imaging techniques such as CT, MRI, PET/CT, and other diagnostic tests like histological examination and endoscopy, as specified in the study protocol. Local recurrence was defined as a recurrence within the pelvis and distant metastasis was defined as a recurrence outside the pelvis, such as in the lung, liver, brain, or bone.

### 2.3 Data extraction and quality assessment

Two researchers extracted information from the included literature using a pre-designed standardized form. The extracted information included the author, publication date, study type, data source, and basic clinical characteristics such as gender, age, number of patients, clinical stage, radiation dosage, preoperative chemotherapy regimen, surgical approach, ACT regimen, follow-up time, and primary outcomes including OS, DFS, CSS, RFS, local recurrence and distant metastasis. If original survival data were not available in the literature, Engauge Digitizer (version 11.3) was performed to extract the oncological outcome data from the Kaplan-Meier curve at the corresponding time point (25).

The quality of retrospective cohort studies was assessed using the Newcastle-Ottawa scale (NOS), which included patient selection (4 points), cohort comparability (2 points), and exposure or outcome assessment (3 points). Scores of 4-6 were considered to be of moderate quality, and scores of 7-9 were considered to be of high quality (26).

The literature search, data extraction, and quality assessment were performed independently by two authors and carefully cross-checked. In case of disagreement, a third author was consulted for active discussion and eventual consensus.

### 2.4 Statistical analysis

The primary outcome of interest in this study was the 5-year OS, DFS, CSS, RFS, distant metastasis, and local recurrence. The data extracted from the studies were summarized and analyzed using Stata version 15 (Stata Corporation, College Station, TX, United States). To assess the impact of ACT on oncological outcomes in patients with ypT0-2N0 rectal cancer, odds ratios (ORs) and 95% confidence intervals (CI) were calculated. Subgroup analysis was performed to evaluate the relationship between ACT and oncological outcomes in rectal cancer patients ypT1-2N0. The random effects model was used for the meta-analysis. The heterogeneity was assessed using Cochran’s Q test and I^2^. Heterogeneity was considered significant if the p-value was lower than 0.05 or I^2^ was greater than 50% (27). Otherwise, there was no significant heterogeneity. Sensitivity analysis (one-by-one exclusion method) was conducted to assess the stability and reliability of the study results in terms of pooled analysis heterogeneity. Funnel plots and Egger’s test were used to evaluate the presence of publication bias in the meta-analyses (28). If significant publication bias was detected, adjusted effect sizes were calculated using the subtractive complementation method. A p-value of less than 0.05 was considered statistically significant.

## 3 Results

### 3.1 Study selection

**Figure 1:**
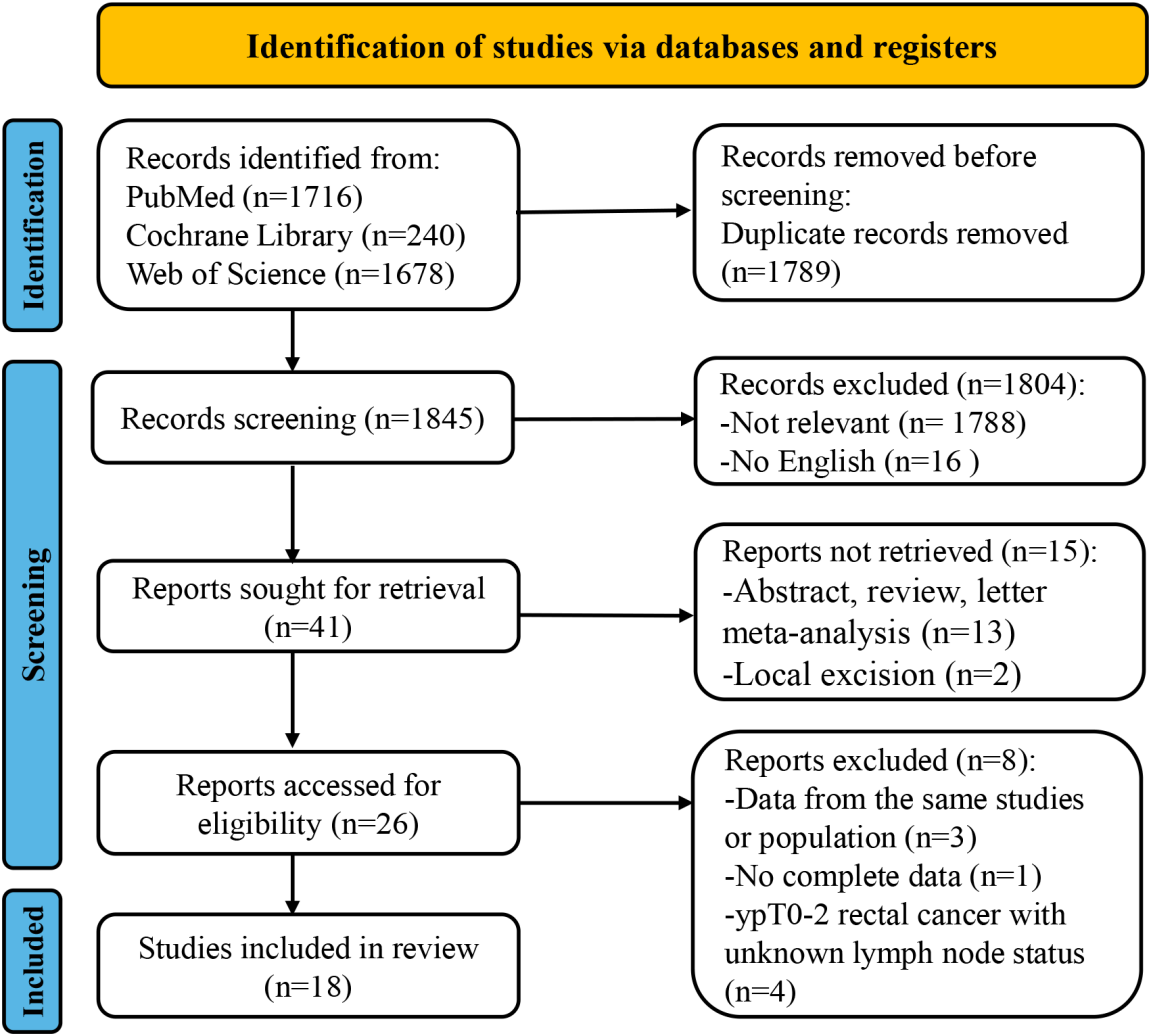
The flow chart of PRISMA.

According to the search criteria, a total of 3634 articles were retrieved from three databases: PubMed (n=1716), Web of Science (n=1678), and Cochrane Library (n=240). After excluding duplicate articles (n=1789), another 1804 articles that did not meet the inclusion criteria were excluded through screening of titles and abstracts. Upon comprehensive evaluation of the full-text articles, four studies were excluded due to data from the same population (n=3), incomplete data (n=1) and ypT0-2 rectal cancer with unknown lymph node status (n=4). Finally, 18 retrospective studies (18–20, 22, 29–42) were included in this meta-analysis. The detailed flowchart is shown in **Figure 1**.

### 3.2 Basic characteristics of the included studies

Studies published between 2009 and 2023 were included in this meta-analysis, comprising nine (19, 22, 30, 34, 36, 38, 40–42) from China and five (29, 32, 33, 35, 39) from Korea. These studies covered a total of 6,566 rectal cancer patients with ypT0-2N0 after NCRT, of which 3,932 were ypT1-2N0. The majority of patients received long-course radiotherapy with a dose of 45-50.4 Gy and fluorouracil-based concurrent chemotherapy, whereas 3,614 rectal cancer patients with ypT0-2N0 also underwent various postoperative ACT regimens, including 5-FU, Capecitabine, CapeOX, and FOLOX. The specific characteristics of the included studies are detailed in **Table 1**.

**Table 1:** The basic characteristics of the included studies.

Study	Publication time	Country	Study type	Period	Sex(F/M)	Age (Obs/ACT)	Radiotherapy (Gy/frequency)	Neoadjuvant chemotherapy	Adjuvant chemotherapy	ypTN stage	Number of patients		Surgery (AR/APR)	Time from RT-Op (weeks)	Follow-up (months)	Outcomes
											ACT	Obs				
Bang et al. (29)	2023	Korea	RCS/Single	2015-2019	NA	71^a^	45-50.4/25-28	Capecitabine	5-FU/Capecitabine/FOLFOX	T0-2N0	48	16	NA	6-8	48.3^a^	OS/RFS
Chen et al. (30)	2015	China	RCS/Single	2002-2009	25/38	56.5^b^	30/10	NA	FOLFOX/FOLFIRI/CapeOX	T0-2N0	24	39	NA	>2	58.5^b^	OS/DFS/DM/LR
Galata et al. (31)	2018	Germany	RCS/Single	1999-2012	28/76	62.9^b^/61.2^b^	50.4/28	XELIRI/CapOX/5-FU/panitumumab	Capecitabine/CapeOX/5-FU	T0-2N0/T1-2N0	54	50	83/21	4-12	68^b^	OS/DFS/DM/LR
Govindarajan et al. (18)	2011	USA	RCS/Single	1993-2003	79/125	60^a^/68^a^	50.4^a^/26^a^	5-FU based	FL/FOLFOX	T0-2N0/T1-2N0	174	30	150/54	4-8	69.6^a^	DM/LR/RFS
Huh et al. (32)	2009	Korea	RCS/Single	1994-2008	8/33	62^b^/55^b^	45-50.4/25-28	FL	FL/UFT/Doxifluridine	T0-2N0	17	24	35/6	6-8	47.6^a^	OS/DFS/DM/LR/RFS
Jung et al. (33)	2014	Korea	RCS/Single	2006-2011	NA	64^a^/54^a^	44^a^/22^a^	5-FU/FL/Capecitabine	5-Fu based	T1-2N0	107	8	104/11	6-8	47.8^a^	DFS
Kuo et al. (34)	2022	China	RCS/TCR/NHIRD	2007-2017	222/498	>60: 152/122	50.4^a^/27^a^	5-FU/Capecitabine/Oxaliplatin/Leucovorin/UFUR	5-FU/Capecitabine/Oxaliplatin/UFUR	T0-2N0/T1-2N0	368	352	501/111	NA	50.6^a^	OS/DFS
Lee et al. (35)	2015	Korea	RCS/Single	1999-2009	NA	NA	50.4/28	Capecitabine	UFT/Doxifluridin/Capecitabine	T0-2N0/T1-2N0	87	38	108/17	6-8	60.5^a^	OS/DFS/LR
Liao et al. (36)	2021	China	RCS/Single	2006-2011	31/79	69.5^b^/62.14^b^	45-50.4/25-28	FL/Capecitabine	FL/Capecitabine	T0-2N0	76	34	98/12	6-8	60^a^	OS/DFS
Liao et al. (1) (22).	2023	China	RCS/SEER	2004-2017	757/1364	<65: 324/955	NA	NA	NA	T1-2N0	455	1666	NA	NA	105^a^	OS/CSS
Lichthardt et al. (37)	2017	Germany	RCS/Single	1992-2013	NA	65^a^	NA	NA	5-FU/Capecitabine/FOLFOX/FOLFIRI	T0-2N0/T1-2N0	37	65	NA	NA	NA	OS
Lu et al. (38)	2018	China	RCS/Single	2005-2014	42/67	59.4^b^/50.4^b^	42-50/21-25	Capecitabine/CapOX	Capecitabine/CapeOX/SOX/FOLFOX	T0-2N0/T1-2N0	58	51	40/69	7.7^a^	50^a^	OS/DM/LR/RFS
Pang et al. (19)	2021	China	RCS/Multi-	2007-2019	261/679	<56: 101/332	50/25	CapeOX/FOLFOX	NA	T0-2N0	705	235	NA	8^a^	40^a^	DFS/CSS/DM/LR
Park et al. (39)	2014	Koera	RCS/Multi-	2004-2009	333/673	65^a^/58^a^	50.4^a^	5-FU/Capecitabine/Irinotecan/Oxaliplatin/oral Fluoropyrimidine/Erbitu	5-FU/Oxaliplatin/Irinotecan	T0-2N0	910	106	850/166	4-12	58^a^	RFS/DM/LR
Voss et al. (20)	2020	USA	RCS/KPSC	2005-2016	NA	59.9^b^	45-55.8	5-FU/Capecitabine/FOLFOX/CapeOX	Capecitabine/5-FU/CapeOX/FOLFOX/Oxaliplatin	T0-2N0/T1-2N0	295	127	NA	6^b^	63^b^	OS/CSS/RFS/DM/LR
You et al. (40)	2014	China	RCS/Single	2003-2010	NA	62^a^/54^a^	46/23	FOLFOX6/CapOX	Capecitabine/CapeOX/FOLFOX6	T0-2N0/T1-2N0	41	16	NA	6^b^	46^b^	OS/DFS/RFS/DM/LR
Zhang et al. (41)	2020	China	RCS/Single	2010-2018	33/88	61.2^b^/55.6^b^	45-50.4/25-28	Capecitabine/CapOX/FOLFOX	Capecitabine/CapeOX/FOLFOX	T0-2N0	90	31	80/41	8.9^a^	40.1^a^	OS/DFS
Zhao et al. (42)	2023	China	RCS/Single	2011-2017	NA	54.7^b^	45-50/25	Capecitabine	CapeOX/Capecitabine	T0-2N0	68	64	NA	8^a^	79^a^	OS/DM

^a^The data was present as median; ^b^The data was present as mean. F, Female; M, male; Obs, Observation; ACT, Adjuvant chemotherapy; AR, Anterior resection; APR, Abdominoperineal resection; RT-Op, Radiotherapy to operation; NA, Not available; RCS, Retrospective cohort study; Single, Single-center; Multi-, Multicenter, FU, Fluorouracil; FOLFOX, Fluorouracil+Leucovorin+Oxaliplatin; XELIRI, Capecitabine+Irinotecan; CapeOX, Capecitabine+Oxaliplatin; FL, Fluorouracil+Leucovorin; UFT, tegafur/uracil; SOX, Oxaliplatin+S-1; OS, Overall survival; DFS, Disease free survival; LR, Local recurrence; DR, Distant recurrence; RFS, Rcurrence-free survival; CSS, Cancer-specific survival.

### 3.3 Quality assessment of studies

The NOS scale was utilized to assess the risk factors and methodological quality of retrospective cohort studies. The median score of the included retrospective cohort studies was 7 (ranging from 6 to 9), indicating an acceptable quality of the cohort studies (**Supplementary Table 2**).

### 3.4 Comparison of oncological outcomes

#### 3.4.1 Overall survival

**Figure 2:**
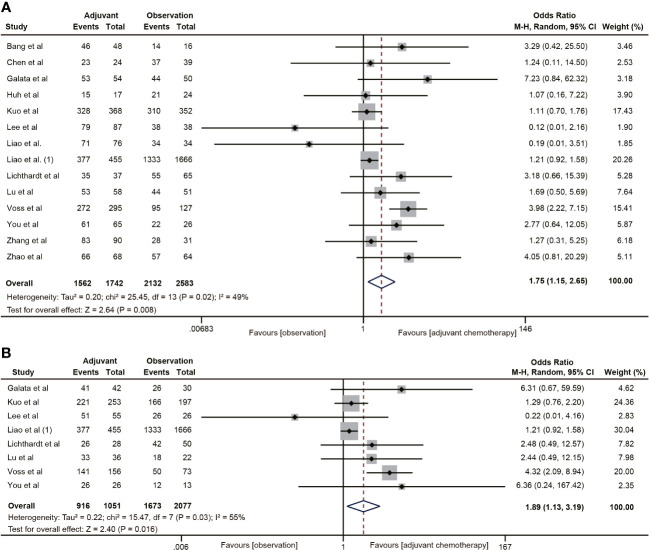
Pooled analysis of the effects of adjuvant chemotherapy on 5-year overall survival. **(A)** ypT0-2N0 rectal cancer patients; **(B)** ypT1-2N0 rectal cancer patients.

A total of 14 (20, 22, 29–38, 40–42) studies involving 4,325 participants reported the effect of ACT on 5-year OS in patients with ypT0-2N0 rectal cancer. The Pooled data showed that ACT significantly improved 5-year OS in patients with ypT0-2N0 rectal cancer compared with the observation group (OR=1.75, 95% CI: 1.15-2.65, P=0.008) (**Figure 2A**). Heterogeneity analysis revealed a moderate level of heterogeneity in the meta-analysis (I^2^ = 49%, P=0.02). Furthermore, subgroup analysis was performed on 8 studies (20, 22, 31, 34, 35, 37, 38, 40) reporting the 5-year OS of ypT1-2N0 rectal cancer patients. The subgroup analysis indicated that ypT1-2N0 rectal cancer was able to benefit from ACT (OR=1.89, 95% CI: 1.13-3.19, P= 0.003). There was a moderate heterogeneity in the study (I^2^ = 55%, P=0.03) (**Figure 2B**).

#### 3.4.2 Disease-free survival

**Figure 3:**
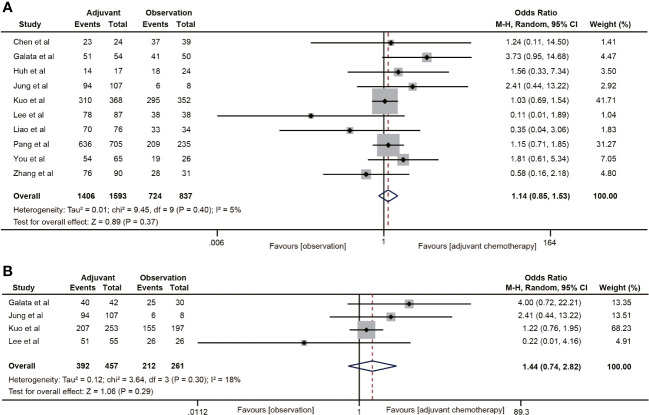
Pooled analysis of the effects of adjuvant chemotherapy on 5-year disease-free survival. **(A)** ypT0-2N0 rectal cancer patients; **(B)** ypT1-2N0 rectal cancer patients.

A total of 10 studies (19, 30–36, 40, 41) reported DFS data for rectal cancer patients with ypT0-2N0. The combined data suggested that the 5-year DFS rate of the ACT group was not higher than that of the non-ACT group (OR=1.14, 95% CI: 0.85-1.53, P=0.37) (**Figure 3A**), and there was no evidence of heterogeneity (I^2^ = 5%, P=0.40). Furthermore, when considering the T1-2N0 subgroups, there was also no statistically significant improvement in 5-year DFS with ACT compared to the observation group (OR=1.44, 95% CI: 0.74-2.82, P=0.29) (**Figure 3B**).

#### 3.4.3 Cancer-specific survival

**Figure 4:**
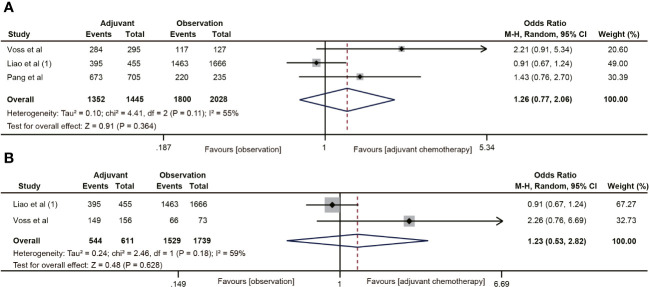
Pooled analysis of the effects of adjuvant chemotherapy on 5-year cancer-specific survival. **(A)** ypT0-2N0 rectal cancer patients; **(B)** ypT1-2N0 rectal cancer patients.

Three (19, 20, 22) of the 18 studies reported 5-year CSS data. A total of 3483 patients with ypT0-2N0 rectal cancer were enrolled in the study. The meta-analysis showed that ACT had no tendency to improve CSS in ypT0-2N0 rectal cancer patients, and there was a moderate heterogeneity (OR=1.26, 95% CI: 0.77-2.06, P=0.364; I^2^ = 55%, P=0.11) (**Figure 4A**). In addition, the benefit of ACT in rectal cancer patients with ypT1-2N0 was also not observed. (OR=1.23, 95% CI: 0.53-2.82, P=0.628) (**Figure 4B**).

#### 3.4.4 Recurrence-free survival

**Figure 5:**
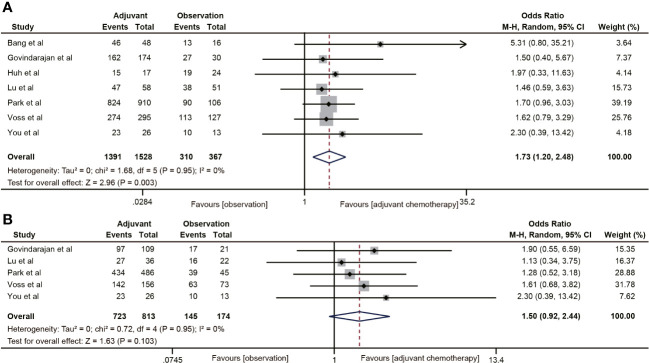
Pooled analysis of the effects of adjuvant chemotherapy on 5-year recurrence-free survival. **(A)** ypT0-2N0 rectal cancer patients; **(B)** ypT1-2N0 rectal cancer patients.

The RFS data was reported in seven studies (18, 20, 29, 32, 38–40). The results indicated that ACT was associated with the 5-year RFS rate of ypT0-2N0 rectal cancer patients (OR=1.73, 95% CI: 1.20-2.48, P=0.003) (**Figure 5A**). No heterogeneity was observed (I^2^ = 0%, P=0.95). Five (18, 20, 38–40) of the six studies included the RFS data of ypT1-2N0 rectal cancer. However, the pooled data did not find ACT beneficial in improving RFS in rectal cancer with ypT1-2N0 (OR=1.50, 95% CI: 0.92-2.44, P=0.103) (**Figure 5B**).

#### 3.4.5 Distant metastasis

**Figure 6:**
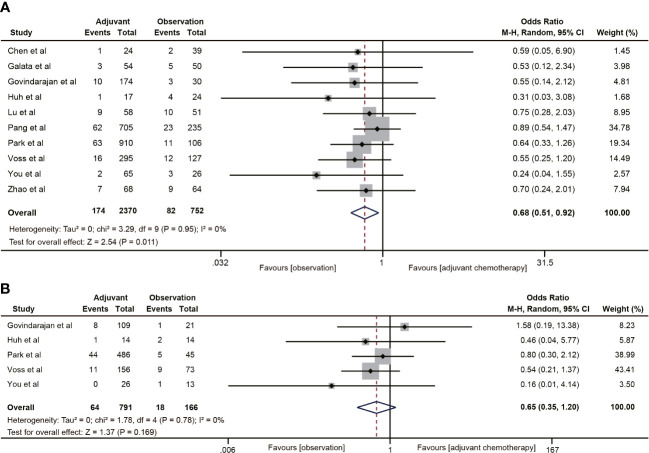
Pooled analysis of the effects of adjuvant chemotherapy on distant metastasis. **(A)** ypT0-2N0 rectal cancer patients; **(B)** ypT1-2N0 rectal cancer patients.

Ten studies (18–20, 30–32, 38–40, 42) reported 5-year distant metastasis for ypT0-2N0 rectal cancer. The pooled data indicated that the distant metastasis rate was lower in the ACT group than in the observation group (OR=0.68, 95% CI: 0.51-0.92, P=0.011) (**Figure 6A**), and the difference was statistically significant (P=0.011). Nevertheless, ACT did not reduce the risk of distant metastasis in ypT1-2N0 rectal cancer (OR=0.65, 95% CI: 0.35-1.20, P=0.169). There was no heterogeneity in the analysis (I^2^ = 0%, P=0.78) (**Figure 6B**).

#### 3.4.6 Local recurrence

**Figure 7:**
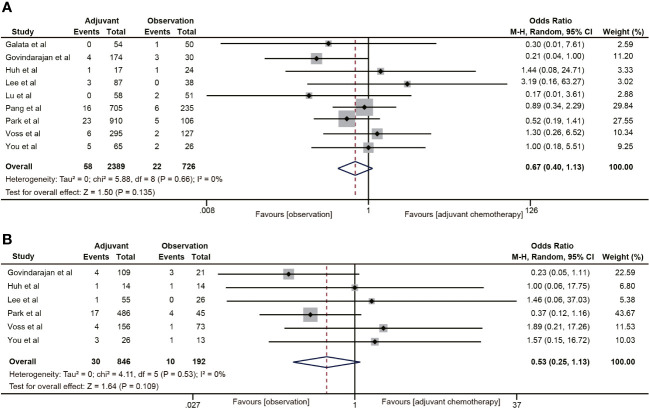
Pooled analysis of the effects of adjuvant chemotherapy on local recurrence. **(A)** ypT0-2N0 rectal cancer patients; **(B)** ypT1-2N0 rectal cancer patients.

Nine (18, 19, 30–32, 35, 38–40) of the 18 studies mentioned 5-year local recurrence data. Although postoperative ACT did not significantly decrease the local recurrence in ypT0-2N0 rectal cancer (OR=0.67, 95% CI: 0.40-1.13, P=0.135) (**Figure 7A**), there was a trend towards lower local recurrence rates in ypT0-2N0 rectal cancer patients receiving ACT. No heterogeneity was observed in the pooled studies (I^2^ = 0%, P=0.66). Moreover, in the subgroup analysis of ypT1-2N0 rectal cancer, postoperative ACT also failed to significantly reduce the local recurrence (OR=0.53, 95% CI: 0.25-1.13, P=0.109) (**Figure 7B**).

#### 3.4.7 Sensitivity analysis and publication bias

The findings suggested that there was a moderate heterogeneity in the pooled data of 5-year OS. Therefore, we performed a sensitivity analysis by excluding studies one by one. The results of sensitivity analysis showed that the re-pooled OR values did not change significantly after excluding studies one by one, and there were no outliers that significantly affected the overall results, indicating that the results of this study are relatively stable (**Supplementary Figure 1**). Funnel plots and Egger’s tests were used to assess publication bias for the primary outcome of 5-year OS. The funnel plot of OS was symmetrical, and the P value of Egger’s test was 0.478, indicating that there was no publication bias among the studies (**Supplementary Figure 2**).

## 4 Discussion

In this meta-analysis, we evaluated the impact of ACT on oncological outcomes in rectal cancer patients who achieved ypT1-2N0 after undergoing NCRT and radical surgery. The pooled data revealed that ACT led to improvements in OS and RFS. Additionally, it was found to reduce the risk of distant metastasis in rectal cancer patients with ypT0-2N0. However, no significant effect on DFS or local recurrence was observed. Subgroup analyses further indicated that ACT also improved OS in rectal cancer patients with ypT1-2N0.

NCRT combined with surgery is now the preferred treatment for rectal cancer patients with transmural (T3/4), lymph node positive and positive circumferential resection margin (3). However, the optimal treatment strategy after NCRT and radical surgery for LARC is still uncertain. According to the NCCN guidelines, all patients with LARC should receive 4-month ACT with fluorouracil alone or combined with oxaliplatin after NCRT, regardless of the postoperative pathological stage (6). This recommendation is based on evidence that postoperative adjuvant chemotherapy or radiotherapy can improve the oncological outcomes of rectal cancer (15). On the other hand, the European Society of Medical Oncology (ESMO) guidelines suggest adjuvant therapy only for pathological stage III or stage II rectal cancer with high-risk factors after NCRT (43). This recommendation stems from several randomized control trials demonstrating the benefit of ACT for patients with pathological stage III or high-risk factors for II colon cancer. However, the evidence for the effectiveness of ACT in rectal cancer patients after NCRT is not as robust as that in colon cancer patients, and ACT is more likely to improve DFS limitedly rather than OS in rectal cancer after NCRT (44–46).

Several retrospective studies have indicated that ACT can extend the DFS and OS of rectal cancer patients following NCRT (7, 13, 47–49). ACT may work by removing microscopic residual cancer cells after surgery, activating immune responses, and inhibiting tumor growth, thereby reducing the risk of recurrence and metastasis and improving treatment outcomes. However, multiple RCTs have not shown any improvement in the oncological outcomes of patients undergoing neoadjuvant radiotherapy/chemoradiotherapy with the use of ACT (9, 23, 50–52). For instance, a randomized controlled trial conducted in Italy randomized 655 patients after neoadjuvant radiotherapy into two groups: one group received 6 cycles of ACT with the de Gramont regimen (fluorouracil and leucovorin), while the other group was observed without any additional treatment. The results revealed that ACT did not provide any advantage in terms of postoperative recurrence (HR=0.977, 95% CI 0.724-1.319) or OS (HR=1.045; 95% CI 0.775-1.410) (23). However, the lack of compliance with postoperative ACT and poor recruitment of subjects in these RCTs may have led to an inadequate evaluation of the efficacy of ACT. Additionally, some studies did not follow the current recommended chemotherapy regimens. A meta-analysis of RCTs also demonstrated that neither single-agent or multi-agent chemotherapy with fluorouracil, nor combination chemotherapy with oxaliplatin-containing regimens, improved OS and DFS in patients with rectal cancer who underwent radical surgery after NCRT (7).

Pathological regression response in rectal cancer after NCRT is also associated with oncological prognosis. Specifically, rectal cancer patients who achieve a pCR after NCRT have a favorable outcome (53). The necessity of ACT for rectal cancer patients with a pCR remains controversial due to potential toxicities associated with this treatment. Several studies using the National Cancer Database have examined the impact of ACT on OS in rectal cancer patients with a pCR (54–57). These studies have indicated that rectal cancer patients with a pCR can benefit from ACT in terms of OS. However, it is important to note that these studies may have overestimated the effectiveness of ACT in pCR rectal cancer. This is because the proportion of patients who did not receive ACT was reported to be higher in these studies compared to the SEER database (58). Additionally, the ACT group in these studies had better characteristics in terms of age and performance status compared to the observation group. On the other hand, several retrospective studies have shown that ACT does not improve overall and DFS, nor does it reduce the risk of local recurrence and distant metastasis in patients with pCR rectal cancer (59–61).

In the context of rectal cancer with good pathological response (ypT0-2N0), the need for ACT remains uncertain. Several studies have indicated that ACT does not have a significant impact on oncological outcomes for rectal cancer patients with good pathological response (18, 22, 23, 34). For instance, the I-CNR-RT trial found that patients who achieved downstaging (ypT0-2N0) had better OS and lower rates of local and distant metastasis, but did not derive any benefit from ACT (23). Similarly, Kuo et al. examined factors influencing oncological survival in patients with ypT0-2N0 rectal cancer and found that ACT only provided limited OS (HR= 1.03, 95% CI, 0.88-1.21) and DFS (HR= 1.05, 95% CI, 0.89-1.224) benefits compared to observation alone (34). However, other studies have shown that ACT can improve survival outcomes for rectal cancer patients with good pathologic response (20, 21, 31, 37). A subgroup analysis of the EORTC 22921 trial, which included rectal cancer patients with clinical negative for lymph nodes at the time of radical resection after NCRT, demonstrated that ACT enhanced OS and DFS in rectal cancer patients with ypT0-2 (HR=0.64, 95% CI, 0.45-0.91). However, this benefit was not observed in rectal cancer patients with a poor response (ypT3-4) after NCRT (21). Galata et al. also showed that ACT improved DFS (94% vs. 86%, P=0.037) and OS (98% vs. 87%, P=0.017) in patients with ypT0-2N0 rectal cancer, particularly among those with ypT2N0 disease (31).

A previous meta-analysis examined the impact of ACT on the oncological outcomes of rectal cancer patients with ypT0-2N0 (62). This analysis included a total of 16 non-randomized controlled studies, 7 of which focused solely on rectal cancer patients with ypT0N0. The findings of this meta-analysis indicated that ACT did not lead to improvements in OS, DFS, local recurrence, or distant metastasis in rectal cancer patients with ypT0-2N0. Furthermore, no benefits of ACT were observed in the subgroup of rectal cancer patients with a pCR and ypT1-2N0. However, our present meta-analysis yielded contrasting results. It revealed that ACT improved OS, RFS, and reduced the risk of distant metastasis in rectal cancer patients with ypT0-2N0. Additionally, there was a tendency for a reduction in local recurrence. Compared with the previous meta-analysis, our study included data from a larger number of studies, resulting in a larger sample size and more representative data. In our meta-analysis, studies that included only rectal cancer patients with pCR were also not included in the meta-analysis. Because pCR rectal cancers have a better oncological prognosis than non-pCR, inclusion of too many pCR rectal cancers in observation group may underestimate the role of adjuvant chemotherapy in ypT0-2N0 stage rectal cancer. However, when we conducted a subgroup analysis on oncological outcomes in rectal cancer patients with ypT1-2N0, our findings indicated that ACT improved OS in these patients, but did not show any association with DFS, RFS, local recurrence, or distant metastasis. The improvement in OS was mainly attributed to a reduction in disease recurrence and cancer-related deaths. However, our meta-analysis did not observe any benefits of ACT on DFS, local recurrence, and distant metastasis in ypT1-2N0 rectal cancer patients.

This might be due to factors such as the age, performance status, and comorbidities of the rectal cancer patients (63–66). Younger age and better performance status are favorable and independent prognostic factors for OS (66). Additionally, ACT is more likely to be performed in younger patients with fewer comorbidities and better performance status due to their higher compliance and tolerance. Among the studies included in our meta-analysis, we also found that patients in the ACT group had lower age and better performance status. To address the issue of low compliance and tolerance of ACT in patients with rectal cancer, a novel treatment approach called total neoadjuvant therapy (TNT) has been proposed. TNT involves intensifying neoadjuvant therapy by administering induction or consolidation chemotherapy concurrently with NCRT (67, 68). In comparison to NCRT alone, TNT has demonstrated several benefits in the cases of LARC. These include enhanced surgical resection rates and organ preservation rates. Additionally, TNT enhances compliance to systemic therapy, reduces chemotherapy toxicity, and increases the proportion of patients who successfully complete chemotherapy, thereby leading to improved rates of pCR (69–71). Furthermore, several RCTs have demonstrated that adding oxaliplatin to fluorouracil-based adjuvant chemotherapy can enhance DFS in patients with rectal cancer following neoadjuvant chemoradiotherapy (52, 72–74). However, the adjuvant chemotherapy regimens mainly consisted of single-agent chemotherapy with fluorouracil or capecitabine in the included studies. Fewer patients with rectal cancer received adjuvant treatment with more modern agents. The lack of individual patient data prevented us from exploring the factors that could affect OS and DFS in rectal cancer patients who had a favorable pathological response. Therefore, it is important to interpret the results of our meta-analysis with caution.

In this meta-analysis, it is important to acknowledge several limitations. Firstly, the majority of the studies were retrospective cohort studies with small sample sizes, which introduced information bias and potential confounding factors. Secondly, despite conducting subgroup and sensitivity analyses, there still existed heterogeneity among the included studies due to variations in sample size, basic characteristics, and treatment processes. The lack of individual patient data prevented exploration of factors influencing OS and DFS in rectal cancer patients with ypT0-2N0, such as age, performance status, NCRT regimen, postoperative complications, and ACT regimen. Consequently, determining the most appropriate ACT regimen and cycles for rectal cancer patients with a good pathological response remains uncertain. Finally, survival hazard ratios are particularly suitable for analyzing time-to-event data. Due to the limited number of reported hazard ratio studies, our meta-analysis focused solely on estimating the impact of ACT on 5-year survival and recurrence rates.

## 5 Conclusions

In conclusion, this meta-analysis suggests that adjuvant chemotherapy may offer benefits in terms of overall survival, recurrence-free survival, and prevention of distant metastasis for rectal cancer patients with ypT0-2N0 after neoadjuvant chemoradiotherapy and radical surgery. However, there is no evidence to demonstrate its effect on disease-free survival and local recurrence. Therefore, further randomized controlled studies are needed to investigate and address these issues, in order to develop the most appropriate therapeutic strategy for rectal cancer patients with a good pathological response.

## Data availability statement

The original contributions presented in the study are included in the article/**Supplementary Material**. Further inquiries can be directed to the corresponding author.

## Author contributions

JY: Conceptualization, Data curation, Formal Analysis, Methodology, Writing – original draft, Writing – review & editing. QD: Data curation, Formal Analysis, Methodology, Writing – review & editing. ZC: Data curation, Methodology, Resources, Writing – review & editing. YC: Data curation, Investigation, Methodology, Writing – review & editing. ZF: Conceptualization, Supervision, Writing – review & editing.
